# Prolonged water limitation shifts the soil microbiome from copiotrophic to oligotrophic lifestyles in Scots pine mesocosms

**DOI:** 10.1111/1758-2229.13211

**Published:** 2023-11-22

**Authors:** Astrid C. H. Jaeger, Martin Hartmann, Rafaela Feola Conz, Johan Six, Emily F. Solly

**Affiliations:** ^1^ Sustainable Agroecosystems Group, Department of Environmental Systems Science ETH Zurich Zurich Switzerland; ^2^ Helmholtz Centre for Environmental Research—UFZ Leipzig Germany

## Abstract

Reductions in soil moisture due to prolonged episodes of drought can potentially affect whole forest ecosystems, including soil microorganisms and their functions. We investigated how the composition of soil microbial communities is affected by prolonged episodes of water limitation. In a mesocosm experiment with Scots pine saplings and natural forest soil maintained at different levels of soil water content over 2 years, we assessed shifts in prokaryotic and fungal communities and related these to changes in plant development and soil properties. Prolonged water limitation induced progressive changes in soil microbial community composition. The dissimilarity between prokaryotic communities at different levels of water limitation increased over time regardless of the recurrent seasons, while fungal communities were less affected by prolonged water limitation. Under low soil water contents, desiccation‐tolerant groups outcompeted less adapted, and the lifestyle of prokaryotic taxa shifted from copiotrophic to oligotrophic. While the abundance of saprotrophic and ligninolytic groups increased alongside an accumulation of dead plant material, the abundance of symbiotic and nutrient‐cycling taxa decreased, likely impairing the development of the trees. Overall, prolonged episodes of drought appeared to continuously alter the structure of microbial communities, pointing to a potential loss of critical functions provided by the soil microbiome.

## Graphical abstract

The soil microbiome is crucial for regulating biogeochemical processes and can thus strongly influence sapling development, especially under drought conditions. In this mesocosm experiment with Scots pine saplings and natural forest soil, prolonged water limitation progressively altered the composition of soil microbial communities, regardless of the recurrent seasons. This points to a potential loss of critical functions the soil microbiome provides.

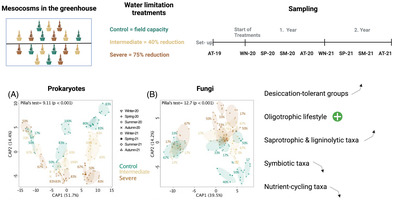

## INTRODUCTION

Climate change is predicted to continue with increased variability in rain and temperature extremes, both inter‐ and intra‐annually (Lee et al., 2021). Although the precipitation projections are variable and region‐dependent, the increase in temperature is consistent and stable across the globe (IPCC, 2022). Thus, an increase in the frequency and intensity of drought periods is expected to be a primary consequence of steadily increasing surface temperatures under future climate change; drought‐induced reductions in soil moisture can potentially generate environmental stress (Manzoni et al., 2012; Schimel et al., 2007), affecting the whole ecosystem, including soil microbial communities and their functions.

Soil microorganisms are responsible for various direct and indirect soil functions essential for plant development (Hartmann & Six, 2022), such as nutrient cycling, plant growth promotion and disease control (Hartmann & Six, 2022; Rosenberg et al., 2014). However, soil moisture reductions potentially affect soil microbial communities that rely on water in soil pores to fulfil their life cycles and functions (Vos et al., 2013). In addition to these direct effects, periods of low soil water content can also indirectly influence soil microbial communities (including prokaryotes and fungi) through alterations in plant growth (Nielsen & Ball, 2015; Tedersoo et al., 2014; Zhou et al., 2020) and soil properties such as pH, salinity or soil organic carbon and nitrogen concentrations (Chodak et al., 2015; Lauber et al., 2009; Yan et al., 2015).

Soil microbes have different coping strategies for fluctuating soil moisture levels (Lennon et al., 2012; Schimel et al., 2007). To tolerate desiccation, microbes can produce protective molecules (Schimel et al., 2007) such as osmolytes, melanin or extracellular polymeric substances (Costa et al., 2018; Naseem et al., 2018; Warren, 2014). Specifically, bacteria endure desiccation by producing cysts, occupying small soil pores, or shifting to dormancy (Lennon & Jones, 2011; Schimel et al., 2007). Unlike prokaryotes, soil fungi are usually less affected by dry conditions—at least in the short term—due to their ability to create and extend exploratory hyphal structures (Barnard et al., 2013; de Vries et al., 2012, 2018; Yuste et al., 2011). Despite these contrasting strategies, the composition of fungal communities can also differ between dry and wet soils (Acosta‐Martínez et al., 2014; Cregger et al., 2012; Crowther et al., 2014; Hawkes et al., 2011; Kaisermann et al., 2015).

Dry environments are often oxygen‐rich but nutrient‐poor and thus termed ‘oligotroph’ (Naylor & Coleman‐Derr, 2018). Hence, oligotrophic microbes are known for being slow growers but able to sustain growth under resource‐poor conditions (Naylor & Coleman‐Derr, 2018; Trivedi et al., 2018). They also tend to be metabolic specialists because they efficiently obtain nutrients from various C sources, including more complex high molecular weight compounds found in plant litter (Fierer et al., 2007; Kurm et al., 2017).

Drought‐induced alterations of soil microbiomes are frequently reported (e.g., Jensen et al., 2003; Seaton et al., 2022; Sheik et al., 2011). However, published findings regarding changes in community composition in response to water limitation are mostly from non‐forested ecosystems, and little is known about how these observations apply to the microbiomes associated with individual tree species (Baldrian et al., 2022).

In the inner‐alpine valleys of Switzerland, an increased frequency and severity of drought events were recorded in the past decades (Bigler et al., 2006; Rebetez & Dobbertin, 2004), leading to diebacks of the local tree species Scots pine (*Pinus sylvestris* L.) (Leuschner & Ellenberg, 2017) essential for the protection against rockfall, avalanches and soil erosion. In addition to the dieback or replacement of mature trees by broadleaf tree species (Dobbertin et al., 2005; Dobbertin & Rigling, 2006; Rigling et al., 2013), high mortality levels during sapling development represent a significant bottleneck to recruitment into the next forest generation (MacAllister et al., 2019). The lack of young trees is especially problematic for forest recovery after extreme events such as pathogen attacks or storms.

Hartmann et al. (2017) and Herzog et al. (2019) found that water‐limiting conditions in a semi‐arid Scots pine forest favour oligotrophic, metabolically versatile and drought‐tolerant taxa. However, there is still a lack of longer‐term observations over multiple time points and across seasons compared to one‐point or end‐point observations in field studies. In particular, studies under controlled conditions are needed to assess the adaptive responses to different soil moisture levels and quantify the related feedback on soil properties and plant development.

A previous study involving a controlled greenhouse experiment with young Scots pine saplings and natural forest soil under different soil water content levels showed that prokaryotic communities were less resistant to water limitation than fungal communities (Jaeger et al., 2023). Moreover, water limitation promoted the proliferation of desiccation‐tolerant taxa and induced a shift in the potential lifestyle of taxa from symbiotic to saprotrophic (Jaeger et al., 2023).

In this study, we harnessed the same mesocosm experiment to test whether water limitation over a prolonged period of 2 years leads to continuous changes in the soil microbiome and how these changes relate to physicochemical soil properties and plant parameters. Moreover, this study was used to identify potential adaptations of soil microbial communities to prolonged water‐limiting conditions and test if effects are linked to the reoccurring seasons. Building on the previous results, we hypothesised that (i) the structure of microbial communities is progressively altered by an extended period of water limitation through continued adaptation to the water‐limiting conditions, (ii) fungal communities remain less sensitive than prokaryotic even to a prolonged reduction in soil water content, (iii) changes in nutrient availability with lower availability of readily available C will induce a shift in microbial communities towards oligotrophic lifestyles and (iv) alterations in substrate quality, for example, changes in soil organic matter C:N ratios will increase the abundance of saprotrophic and ligninolytic taxa.

## EXPERIMENTAL PROCEDURES

### Experimental set‐up

Preparation of the plant–soil systems (subsequently referred to as ‘mesocosms’) was conducted as previously described in Jaeger et al. (2023) and Solly et al. (2023). In brief, 18 three‐year‐old Scots pine (*P. sylvestris* L.) saplings were planted with homogenised natural forest soil in individual pots (32 cm height × 69 cm diameter, 100 L volume) in two greenhouses at the Research Station for Plant Sciences Lindau (ETH Zurich, Switzerland). The forest soil was collected from a mature xeric forest stand dominated by Scots pine trees in the Rhone valley (Pfynwald, Canton Valais, Switzerland, 46°18′16.1" N, 7°36′44.8″ E, 600 m a.s.l.). For 3 months, the mesocosms were watered twice weekly with 2 L of local rainwater reaching a volumetric water content (VWC) of approximately 30% (close to field capacity, which was ~35% VWC, with a pF of 1.8). In January 2020, the mesocosms were assigned to three different irrigation treatments in a randomised design to minimise spatial effects (i.e., variability in light availability). The three levels of irrigation were: Sufficient water supply (control; 30% VWC; *n* = 6), decreased amount of water (intermediate; 40% reduction in VWC of control; *n* = 6), and water stress (severe; 75% reduction in VWC of control; *n* = 6). The intermediate treatment represents the maximum forecasted deviation of precipitation from the normal under a future climate in Southern Switzerland (period: 2081–2100) (CH2018, 2018; Fischer et al., 2022). The soil water content in the severe treatment was kept at a level at which the trees received a minimum of water so as not to suffer from permanent damage and to maintain vitality. The greenhouse temperature was controlled to account for seasonal changes in temperature according to climatological data measured at the MeteoSwiss meteorological station of Sion (Canton Valais, Switzerland) (Table S1), close to where the forest soil used for the mesocosms was collected. The humidity in the greenhouse ranged between 50% and 70% throughout the experiment (Table S1). Greenhouse temperatures and humidity were constantly monitored. The mesocosms were equipped with soil sensors measuring VWC and soil temperature every 60 min (Teros 11, Teros 21, Meter Group, Pullman, WA, USA).

### Soil sampling

Bulk soil sampling was performed once every season for 2 years (*n* = 8) in all pots (*n* = 18) at different stages of plant growth and temperature settings with a slide‐hammer corer (5.5 cm diameter) to a depth of ~25 cm. The holes in the mesocosm systems produced by the sampling were filled with sterilised sand to minimise soil structure disruption. In the following, the sampling time points will be termed ‘seasons’ referring to successional stages of tree development and named ‘winter‐20’, ‘spring‐20’, ‘summer‐20’, ‘autumn‐20’, ‘winter‐21’, ‘spring‐21’, ‘summer‐21’ and ‘autumn‐21’. After sampling, the soil samples were kept cool, transported to the lab and immediately stored at 4°C before further processing.

### Physicochemical soil characteristics

The fresh soil samples were sieved to 4 mm, and large organic residues were removed manually. A subsample (0.250 g of soil) for DNA extraction was stored at −20°C until further analysis. Fresh soil samples were used to analyse gravimetric water content (GWC, 10 g of soil), inorganic nitrogen (NH_4_^+^, NO_3_^−^, 10 g of soil) and K_2_SO_4_ extractable organic carbon (EOC, 12.5 g of soil). The rest of the soil samples were dried at 40°C to constant weight and sieved to 2 mm for measurements of soil pH, total carbon (TC), inorganic carbon (IC), organic carbon (C_org_), and total nitrogen (TN).

The GWC of the soil was assessed by weighing a subsample of 10 g of the soil before and after drying at 105°C for at least 48 h. Ammonium (NH_4_^+^) and nitrate (NO_3_^−^) concentrations of the soils were determined by extraction of 10 g soil with 50 mL 2 M KCl solution in a 1:5 soil:solution ratio and shaken for 1 h at 180 rpm. After filtering through a 150 nm ashless filter paper (Whatman No. 42), the extract was stored at −20°C until further analyses. NH_4_^+^ and NO_3_^−^ concentrations were determined colorimetrical with a spectrophotometer v‐1200 (VWR, Radnor, PA, USA) following Forster (1995) for NH_4_^+^ and Doane and Horwáth (2003) for NO_3_^−^. EOC was extracted with 12.5 g of soil and 50 mL of 0.5 M K_2_SO_4_ in a 1:4 soil:solution ratio and shaken for 1 h at 180 rpm. After filtering through a 150 nm ashless filter paper (Whatman No. 42), the extract was stored at −20°C until further analysis. The EOC concentration of the extracts was determined after 1:5 dilution with MiliQ‐water and acidification with 100 μL of 2 M HCL using the combustion catalytic oxidation method of a TOC‐analyser (TOC‐L series, Shimadzu, Kyoto, Japan). Soil pH was measured in a 1:2.5 solution containing 10 g of dried soil and 25 mL of 0.01 M CaCl_2_ solution. Samples were shaken horizontally at 180 rpm for 1 h and stored overnight to allow sedimentation before measurement with a pH meter (VWR, Radnor, PA, USA). TC and TN concentrations were determined on milled soil samples with an elemental analyser LECO 628 (LECO, St. Joseph, MI, USA). Inorganic carbon (IC) was measured with the pressure‐calcimeter method following Sherrod et al. (2002). The organic carbon (C_org_) was calculated as C_org_ = TC − IC and used to determine C_org_/TN, referred to as C:N ratio.

### Tree growth and photosynthesis

Every month, tree growth was monitored by measuring tree height and stem diameter as proxies of aboveground plant productivity. The height, including the buds, was measured, and the increment was calculated as tree growth. The diameter was measured at two angles, and the mean was taken. Here, the seasonal increment was calculated as radial growth. The needle litter was collected each season on a polyethylene net (mesh size 3 × 3 mm) placed above the soil surface. The collected needle litter was weighed, dried for 1 week at 40°C, and redistributed on the soil surface according to the average needle fall for each of the three irrigation treatments. After the first growing season, root biomass was collected each season. While sieving the soil, roots were carefully picked out manually, and fine roots with a diameter <2 mm were washed with Milli‐Q water to remove any adhering soil particles. Dead roots were removed based on qualitative visual characteristics such as colour and breakability following Solly et al. (2013). Living fine roots were dried at 70°C, and their dry weight was assessed as living root biomass. As a proxy of plant vitality, light‐saturated photosynthesis was measured each season for 1 year (spring‐20 to spring‐21) to assess the maximum rate at which needles can fix carbon during photosynthesis (Johnstone et al., 2013). Here, 25 south exposed needles were enclosed in the 2 × 3 cm chamber of the LI‐COR LI‐6400 (LI‐COR Biosciences, Lincoln, NE, USA), and leaf gas exchange (A_net_) was measured under 400 μmole mol^−1^ CO_2_, 1000 PAR, local humidity and temperature, and a stomatal ratio of 1.

### Microbial gene abundance

The DNeasy PowerSoil Pro Kit (Qiagen, Hilden, Germany) was used for DNA extraction from 0.250 g fresh soil according to the manufacturer's instructions using the QIAcube System (Qiagen). The quality and quantity of extracted DNA were measured via UV/VIS spectrophotometry with the QIAxpert System (Qiagen). Extracted DNA was diluted to 2 ng/μL to reduce inhibition. The abundance of taxonomic groups—bacteria and archaea, hereafter termed prokaryotes, and fungi—was assessed using a SYBR Green‐based qPCR approach, as previously reported in Jaeger et al. (2023). Potential amplification inhibition induced by unintentional co‐extraction of contaminants was tested across all samples by spiking pGEM‐T plasmid (GenBank® Accession No. X65308; Promega, Madison, WI, USA) into the soil DNA at equimolar concentrations in all samples and amplifying a region on the plasmid using specific primers SP6 and T7 (Microsynth, Balgach, Switzerland). The qPCR standards were produced from purified PCR products obtained by pooling DNA from randomly selected samples. For all reactions, standard curves were obtained from diluted standards with concentrations ranging from 10^−2^ to 10^−8^ ng of DNA template, and a negative control containing ddH_2_O was used. The selected primers (Microsynth) 515F (Parada et al., 2016) and 806R (Frey et al., 2016) targeted the 16S rRNA gene (V4 region). The primers FF390 (nu‐SSU‐1334‐5′) and FR1 (nu‐SSU‐1648‐3′) (Vainio & Hantula, 2000) were chosen to target the 18S rRNA gene (V7–V8 region). All qPCRs were performed in technical triplicates in a thermocycler CFX96 Touch Real‐Time System (Bio‐Rad Laboratories, Hercules, CA, USA). Melting curves were generated to verify the amplification specificity. The results were documented and analysed using the CFX Maestro software (Bio‐Rad Laboratories). The qPCR efficiency was 90%–100% with an *R* ^2^ > 0.99 for all runs.

### Microbial community structure

DNA metabarcoding of ribosomal markers was used to investigate changes in prokaryotic and fungal community structures, as described in Jaeger et al. (2023). The extracted DNA was diluted to 20 ng/μL to reduce inhibition. PCR amplification of the prokaryotic 16S rRNA gene (V3–V4 region) was performed using the primers 341F and 806R (Frey et al., 2016). The fungal ITS2 region of the *rrn* operon was targeted using the primers ITS3ngs and ITS4ngs (Tedersoo & Lindahl, 2016). PCR amplification was carried out in technical triplicates, and triplicates were pooled before sequencing. Pooled PCR products were sent to the Functional Genomics Center Zurich (FGCZ, Zurich, Switzerland) for indexing PCR. Indexed PCR products were purified, quantified and pooled in equimolar ratios. To achieve optimal read count distributions across all samples, pre‐sequencing on the Illumina MiniSeq platform (Illumina, San Diego, CA, USA) was performed to inform library re‐pooling. Final sequencing was executed on the Illumina MiSeq platform (Illumina) using the v3 chemistry for PE300 reads.

### Bioinformatics

Sequencing data were processed using a customised bioinformatics pipeline primarily based on VSEARCH v2.22.1 (Rognes et al., 2016). Primers used in PCR were trimmed with CUTADAPT v4.1 (Martin, 2011), allowing for one mismatch. Bowtie2 v2.4.5 (Langmead & Salzberg, 2012) was used to filter for PhiX contamination by aligning the reads against the PhiX genome (accession NC_001422.1). The *fastq_mergepairs* function in VSEARCH was used to merge trimmed paired‐end reads, and the *fastq_filter* function was applied for quality filtering with a maximum expected error of 1 (Edgar & Flyvbjerg, 2015). Sequences were dereplicated using the *derep_fulllength* function in VSEARCH and delineated into amplicon sequence variants (ASVs) applying the UNOISE algorithm (Edgar, 2016b) of VSEARCH with an alpha of 2 and a minsise of 8. The UCHIME2 algorithm (Edgar, 2016c) implemented as the *uchime3_denovo* function in VSEARCH was used to identify and remove potentially chimeric ASV sequences. The remaining ASV sequences were tested for ribosomal signatures using Metaxa2 v.2.2.3 (Bengtsson‐Palme et al., 2015) for the 16S rRNA gene and ITSx v.1.1.3 (Bengtsson‐Palme et al., 2013) for the ITS2 sequences, and non‐matching sequences were discarded. The final ASV table was generated by mapping the quality‐filtered reads of each sample against the verified ASV sequences executing the *usearch_global* algorithm implemented in VSEARCH with the following settings: maxhits 1, maxrejects 100, maxaccepts 0 and a minimum identity of 97%. Verified ASV sequences were taxonomically classified by running the SINTAX algorithm (Edgar, 2016a) of VSEARCH against the SILVA v.138 database (Quast et al., 2013) for the 16S rRNA gene sequences and the UNITE v.8.3 database (Abarenkov et al., 2010; Nilsson et al., 2019) for the ITS2 sequences, using a bootstrap cut‐off of 0.8. ASVs not assigned at the domain level of bacteria, archaea or fungi, and ASVs assigned to organelle structures (chloroplasts and mitochondria) were removed from the ASV table. The raw sequences were deposited in the European Nucleotide Archive (ENA) under the accession numbers PRJEB53192 and PRJEB61158.

Each prokaryotic ASV was classified into oligotroph or copiotroph lifestyle based on *rrn* copy numbers using the RDP classifier (Wang et al., 2007) and the NCBI taxonomy integrated within the *rrn* operon database (Stoddard et al., 2015). Here, we have classified taxa into copiotrophs by using a cut‐off level of ≥5 and ≥2 gene copies and into oligotrophs with lower gene copy numbers at cut‐off levels of <5 and <2, according to Klappenbach et al. (2000) and Bledsoe et al. (2020). The classification was performed at the lowest possible taxonomic rank, provided in the *rrn* operon database.

For inferences about the potential lifestyle of the taxa, Faprotax v1.2.5 (Louca et al., 2016) and FUNGuild v.1.1 (Nguyen et al., 2016) for prokaryotes and fungi, respectively, were used in conjunction with literature research.

### Statistical analyses

All statistical analyses were computed in R Version 4.2.2 (R Core Team, 2023) using RStudio Version 2022.07.0 (RStudio Team, 2022), and plots were created using *ggplot2* v.3.4.0 (Wickham et al., 2023) unless indicated otherwise. A *p*‐value <0.05 was considered significant for all tests unless mentioned otherwise. The data were fitted to linear mixed effect models (lme) to test the effect of the treatment and the time point (season) on the investigated soil parameters, tree parameters, microbial gene abundance and the copiotroph‐to‐oligotroph ratio. Here, the *lme* function of the package *nlme* v. 3.1‐160 (Pinheiro et al., 2022) was applied using the restricted maximum likelihood method (REML) (Meyer, 1989). Treatment (control, intermediate, severe, *n* = 6) and time point (season, *n* = 8) were used as factor variables with interaction. The greenhouse (Greenhouses 1 and 2) and the pot number (Pots 1–18) were assumed to be a nested random effect. The data were transformed when visual inspection of residual diagnostic plots revealed that the data deviated from the assumption of normality. The functions *log* (for VWC, TN, EOC, copiotroph:oligotroph ratio), *log10* (for NO_3_^−^, 16S gene copies, 18S gene copies, root biomass, needle litter) or *sqrt* (for C:N ratio, NH_4_^+^) were applied. The data were back‐transformed for further tests using the *ref_grid* function of the package *emmeans* v.1.8.2 (Russell et al., 2022). Estimated marginal means with the *emmeans* function were calculated to test the effects of treatment and time point (season). Pairwise comparisons were performed by the *contrast* function of the same package. The function *cld* of the package *multcomp* v.1.4‐20 (Hothorn et al., 2023) was applied to identify and display significant differences between groups.

Sequencing depth was investigated using barplots and rarefaction curves (Figure S5A,B) with the *rarecurve* function in the *vegan* package v.2.6‐4 (Oksanen et al., 2022). Changes in α‐diversity (observed richness, Pielou's evenness and Shannon diversity) and β‐diversity (Bray–Curtis dissimilarity) were calculated from 100‐fold iteratively subsampled and square‐root transformed ASV count tables (Hemkemeyer et al., 2019; Martiny et al., 2017) to account for differences in sequencing depth. Here, the functions *rrarefy*, *specnumber*, *diversity* and *vegdist* in *vegan* were applied. The effects of treatment and time point (season) on α‐ and β‐diversity were assessed using univariate or multivariate permutational analysis of variance (PERMANOVA; Anderson, 2001) and permutational analysis of multivariate dispersions (PERMDISP; Anderson, 2006) with 999 permutations, as implemented in the *adonis2* and *betadisper* functions of *vegan*. Pairwise comparisons between factor levels were performed using the *pairwise.perm.manova* function from the package *RVAideMemoire* v.0.9‐81‐2 (Hervé, 2023). The function *meandist* of the *vegan* package was used to calculate the mean within‐cluster dissimilarities of each treatment and the mean of the between‐cluster dissimilarities (compared to the control treatment) for each time point based on the Bray–Curtis dissimilarity. Similarly, the standard error was calculated. To test for significant differences between and within block dissimilarities, pairwise comparisons were computed with the *pairwise.perm.manova* function.

Differences in β‐diversity were assessed by unconstrained ordination using principal coordinate analysis (PCO) (Gower, 2015) with the *cmdscale* function (Figure 7SA,B). Constrained ordination was performed using canonical analysis of principal coordinates (CAP) (Anderson & Willis, 2003) implemented as the *CAPdiscrim* function in the *BiodiversityR* package v.2.14‐4 (Kindt, 2022), with 999 permutations, setting the factors treatment and time point (season) as constraining factors. Here, the CAP reclassification success rate quantitatively estimates the degree of discrimination between treatment groups. The effects of measured physiochemical soil properties and plant parameters on microbial communities were obtained using the PERMANOVA test. Additionally, factors labelled as significant in the PERMANOVA test were further used as a constraining factor in building a parsimony model executing the function *ordistep* in *vegan*, and the significant factors were displayed by distance‐based redundancy analysis (db‐RDA) (Legendre & Andersson, 1999), using the *dbrda* function in *vegan*. The response of individual taxonomic groups from phylum to genus level towards water limitation was assessed using univariate PERMANOVA based on Euclidean distances via the *adonis2* function with 999 permutations on aggregated data at each taxonomic level, that is, summing up the read counts of ASVs assigned to the same taxonomic group (Supporting Information). The *qvalue* function of the R package *qvalue* v.2.28.0 (Storey & Bass, 2022) was used to adjust for multiple testing by calculating *q‐values* (Storey & Tibshirani, 2003) and *q‐values <0.05* were considered significant, and *q‐values <0.1* as marginally significant. The data were scaled (z‐transformed) to calculate and visualise changes in relative abundance compared to the control treatment. Pairwise comparisons between the water limitation and control treatments were performed and corrected (Benjamini & Hochberg, 1995) with the *pairwise.wilcox.test* to identify significant differences. Heatmaps were generated using the *heatmap.2* function of the package *gplots* v.3.1.3 (Warnes et al., 2022) based on z‐transformed data. Cluster analysis based on the Ward method was performed to group phyla with similar sample structures.

The relative abundance of copiotroph and oligotroph ASVs was summed for each soil sample to calculate the copiotroph‐to‐oligotroph ratio within a prokaryotic community. To test the effect of the treatment and time point (season) and to detect significant differences, the *emmeans* and *contrast* functions were used as previously described.

A subset of the data for the second year (winter‐21 to autumn‐21) was created. This subset was used for correlation‐based indicator species analysis (De Cáceres et al., 2010; De Cáceres & Legendre, 2009) performed on significant ASVs from the PERMANOVA analysis to determine the association strength of each ASV with a time point (season) and treatment or a combination therefore. The function *multipatt* of the package *indicspecies* v.1.7.12 (De Cáceres et al., 2022) with a max.order of 10 and the function “r.g” (to correct for unequal group sizes) was used. *p*‐Value adjustments for multiple comparisons were performed using the false discovery rate correction according to Storey (2002), and associations were considered significant at *q‐value* <0.05. Bipartite association networks were created using Cytoscape v.3.9.1 (Shannon et al., 2003). Following Hartmann et al. (2015), the bipartite networks were generated by using the time point (season) and treatment combination as source nodes and the ASVs as target nodes, with edges corresponding to positive associations of ASVs with time point and treatment combinations as obtained from the indicator species analysis. Networks split by the domain were constructed using the Allegro Fruchtermann‐Reingold algorithm (Fruchterman & Reingold, 1991), with edges weighted according to the association strength.

## RESULTS

### Water limitation treatments

Following the start of the three irrigation treatments (control, intermediate, severe), the VWC of the soils consistently differed among the treatments for all time points (*p* < 0.0001, Table S2, Figure S2). Throughout the experiment, the VWC of the mesocosms significantly varied across time points (seasons) (*p* < 0.0001, Table S2). The highest water content values were measured in spring‐20 for all treatments, whereas the lowest values were obtained in spring‐21 and summer‐21 (Figure S2).

### Soil microbial communities

Water limitation did not affect prokaryotic and fungal abundance (Table S2, Figure S6A,B). However, the time point (season) influenced the abundance of prokaryotic and fungal gene copies (*p* < 0.0001, Table S2). For prokaryotes, larger estimated copy numbers were detected in summer‐20 and for fungi in summer‐20 and between spring‐21 to autumn‐21 (Figure S6A,B).

Metabarcoding yielded 6′912′947 16S rRNA gene sequences delineated into 25′476 ASVs, and 6′126′576 ITS2 sequences delineated into 3′128 ASVs were obtained across 143 samples.

Prokaryotic α‐diversity (examined by observed richness, Pielou's evenness and Shannon diversity) was significantly (*p* ≤ 0.002) affected by the treatment (Table S3). After the start of the water‐limiting treatments, the prokaryotic α‐diversity differed between the treatments at each time point (season) (Figure S6C–E). While α‐diversity was higher under the severe treatment in spring‐20, at all other times, it remained higher under the control treatment (Figure S6C–E). Furthermore, the time point (season) significantly affected prokaryotic α‐diversity (*p =* 0.001, Table S3). Alpha diversity peaked in all treatments in summer‐20 and spring‐21 (Figure S6C–E). Moreover, the interaction of treatment and time point (season) affected Pielou's evenness and Shannon diversity (*p* ≤ 0.003, Table S3).

Fungal observed richness (*p* = 0.001) and Shannon diversity *(p* = 0.048) but not Pielou's evenness were affected by treatment (Table S3). Overall, after spring‐20, α‐diversity remained marginally higher under the severe and intermediate treatment than the control. However, this difference was only significant in spring‐20 as well as in summer‐21 and autumn‐21 for the observed richness (Figure S6F). The time point also significantly affected fungal α‐diversity (*p* ≤ 0.006, Table S3). Pielou's evenness and Shannon diversity remained comparable in the first year (2020) and decreased only slightly in the second year, showing the highest variability in summer‐21 (Figure S6G,H). The observed richness marginally decreased throughout the experiment, showing a significant (*p* = 0.048) interaction between the time point (season) and treatment (Table S3).

Water limitation altered prokaryotic and fungal (*p* = 0.001, Table S3) β‐diversity, explaining 6% and 3% of the variance, respectively (Table S3). In comparison, the effect of the timing (season) on β‐diversity was more substantial, explaining 13% and 15% of the variance, respectively (Table S3). For prokaryotes, the treatment effects depended on the time point (*p* = 0.001), whereas no treatment‐by‐season interaction was found for fungi (Table S3).

**FIGURE 1:**
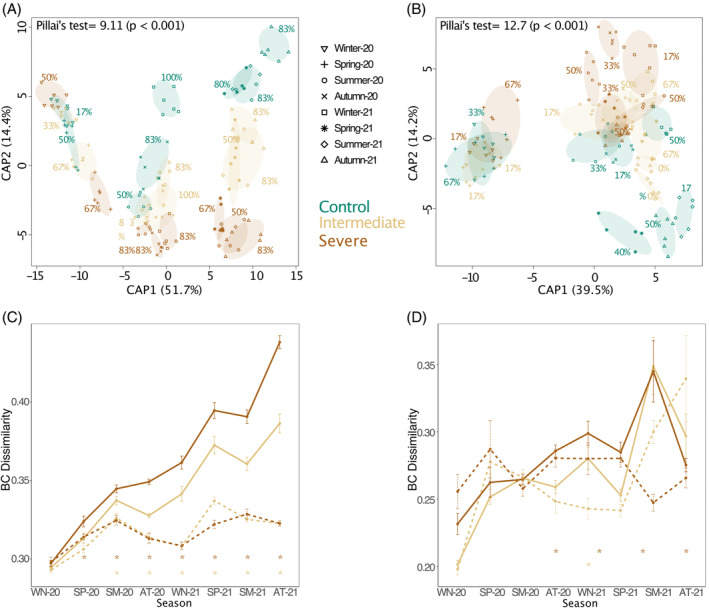
Soil microbial community structures across treatments and seasons and within‐ and between‐cluster Bray–Curtis dissimilarities across treatments. (A,B) Canonical analysis of principal coordinates (CAP) constraining differences in prokaryotic (A) and fungal (B) community structure by irrigation treatment and time point (season). Axis CAP1 represents the time effect, whereas axis CAP2 represents the treatment effect. The CAP reclassification success rates providing a quantitative estimation of the degree of discrimination between the groups are provided next to each group. The CAP equivalent to Pillai's trace test (with *p*‐value in brackets) indicating the overall effect size is provided at the top of the plot. (C,D) Within‐ and between‐cluster Bray–Curtis dissimilarities across irrigation treatments and time points (seasons: WN, winter; SP, spring; SM, summer; AT, autumn). Mean of the within‐cluster dissimilarities (dashed line) and mean of the between‐cluster (solid line) dissimilarities (as compared to the control treatment) for the intermediate and severe treatment at each time point (season) based on the Bray–Curtis dissimilarity. Panel C represents prokaryotic communities, whereas D represents fungal communities. Error bars indicate the standard error of the mean. Significant differences (*p* < 0.05) between between‐cluster and within‐cluster dissimilarities are marked with an asterisk as obtained by pairwise comparisons.

After the start of the experimental treatments, each combination of time point (season) and treatment group harboured distinct prokaryotic microbial communities (Figure 1A,B). The reclassification success rates—as a measure of the degree of discrimination—increased in the second year (Figure 1A,B). However, generally lower reclassification rates were observed for fungal communities, especially under intermediate and severe water limitations, indicating smaller differences in fungal community structures across treatments (Figure 1B).

Under intermediate and severe water‐limiting conditions, the Bray–Curtis dissimilarity of prokaryotic communities between the two water‐limitation treatments and the well‐watered control increased over time, with the most considerable differences observed at the end of the 2‐year experiment (Figure 1C). Concurrently, between‐cluster dissimilarities differed significantly from the within‐cluster dissimilarities, which remained comparable throughout the experiment. In contrast, fungal communities in mesocosms under intermediate water limitation showed only in winter‐21, a significant difference in between‐ and within‐cluster dissimilarities (Figure 1D). Under severe water limitation, a significant difference in between‐ and within‐cluster dissimilarities was found for autumn‐20 and winter‐21 as well as for summer‐21 and autumn‐21 (Figure 1D). Under both water‐limiting treatments, the seasonal within‐cluster dissimilarity of fungal communities was more variable compared to the prokaryotic communities (Figure 1C,D).

### Taxa affected by prolonged water limitation

**FIGURE 2:**
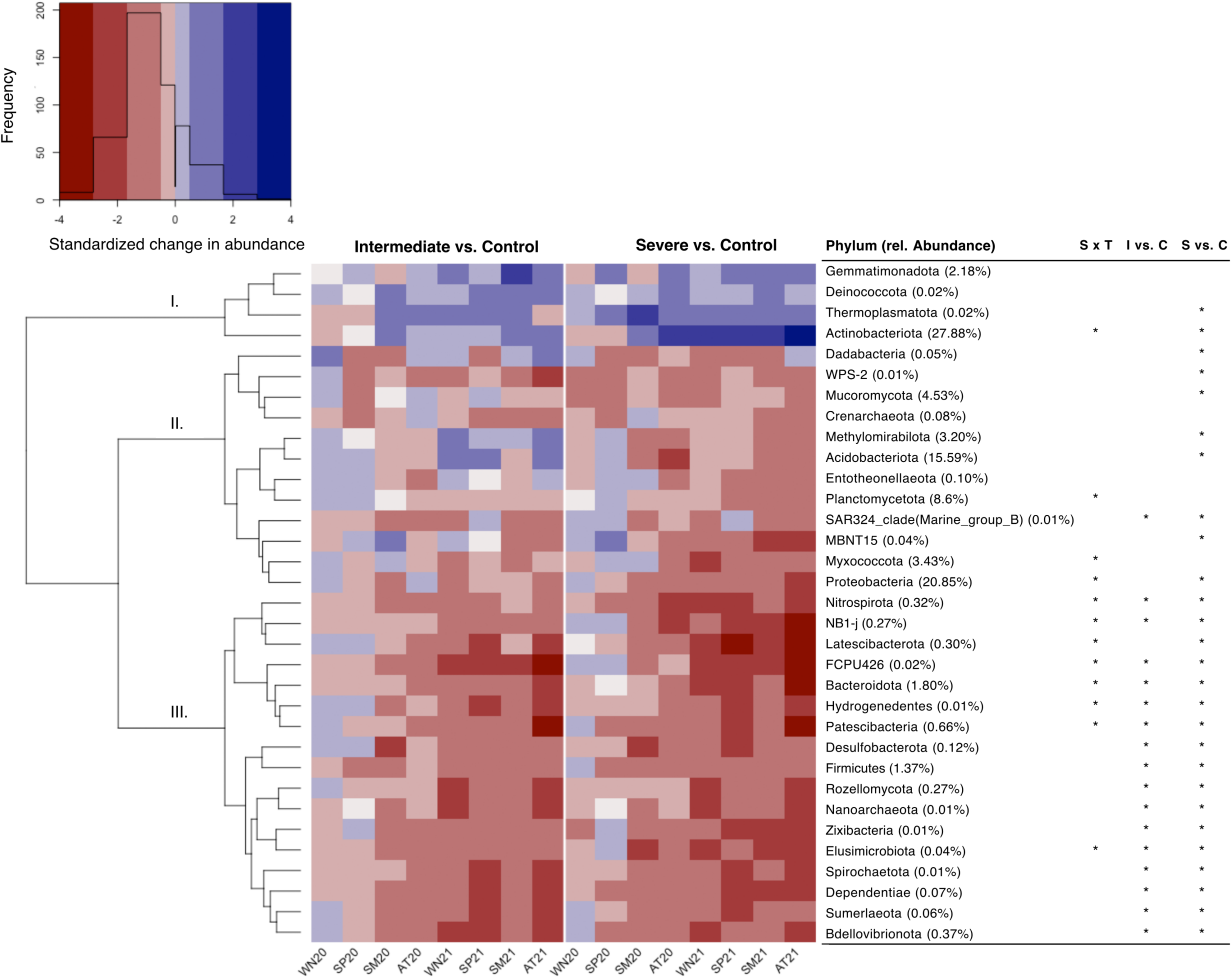
Heatmap showing standardized changes in relative abundance of phyla across seasons compared to the control. Data were scaled (z‐transformed) to compare groups of different abundances, and only phyla with a significant response to the treatment effect (*q* < 0.05) are displayed. Changes in relative abundance under the intermediate and severe treatment compared to the control treatment were calculated. Cluster analysis based on the Ward method was performed to group phyla with similar sample structures. The three identified clusters are termed as follows: I = benefitting, II = tolerant, III = susceptible. The overall relative abundance of each phylum is provided in parenthesis after the name. A significant response (*q* < 0.05) to the combination of the treatment and time (season: WN, winter; SP, spring; SM, summer; AT, autumn) is indicated with an asterisk. Significant results (*p* < 0.05) of pairwise comparisons between the treatments are indicated with an asterisk.

After correction for multiple testing, 5037 out of 25,476 (19.8%) prokaryotic ASVs but only 52 out of 3128 (1.7%) fungal ASVs were observed to be significantly (PERMANOVA; *q <* 0.05) influenced by water deficit. These ASVs corresponded to 31 prokaryotic and 2 fungal phyla (Figure 2). Throughout the observation period, a gradual decrease in relative abundance under intermediate and severe water deficit compared to the control was observed for most of the responsive prokaryotic phyla and the two responsive fungal phyla (Mucoromycota and Rozellomycota). Cluster analysis showed a clear separation of three main clusters according to the response to the water limitation treatments (Figure 2).

The fungal phylum Mucoromycota increased in relative abundance under intermediate water deficit compared to the control but only in autumn‐20 and spring‐21 (Figure 2). The bacterial phyla Deinococcota, Gemmatimonadota and the archaeal phylum Thermoplasmatota increased in relative abundance under water limitation but only towards the end of the observation period. Acidobacteriota, Dadabacteria and Methylomirabilota could tolerate intermediate water limitation but decreased with prolonged severe water limitation (Figure 2). The bacterial phylum Actinobacteriota was the most abundant (28%) and showed a significantly higher relative abundance under water limitation compared to the control. Out of the 31 responsive prokaryotic phyla, 12 phyla, such as Acidobacteriota, Proteobacteria and Nitrospirota, showed a significant response to the interaction of treatment and time point (season) (Figure 2).

ASVs sensitive to water limitation, assigned at a genus level, belonged to different major phyla. Among the most abundant genera were *Umbelopsis* (3.7%, Mucoromycota), *Solirubrobacter* (1.6%, Actinobacteriota) and *Gaiella* (1.3%, Actinobacteriota). At the genus level, most of the significantly affected taxa either gradually decreased or increased in abundance compared to the control (Figure S8).

**FIGURE 3:**
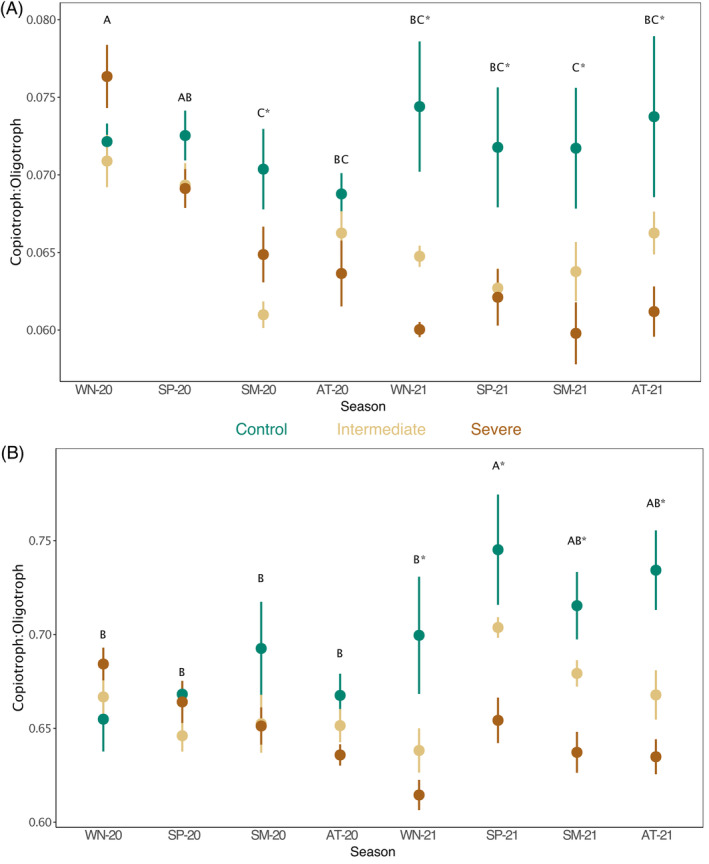
Bacterial copiotroph to oligotroph ratio based on *rrn* copy number estimations. The copiotroph‐to‐oligotroph ratio was calculated based on the cumulative relative abundance of either oligotroph or copiotroph‐classified bacterial ASVs. Panel (A) shows the ratio based on a cut‐off level of <5 *rrn* copies for oligotroph taxa. Panel (B) shows the ratio based on a cut‐off level of <2 *rrn* copies for oligotroph taxa. Letters indicate differences between time points (seasons) obtained by estimated marginal means of linear mixed effect models. The statistical significance of planned contrasts between treatments at each time point (seasons: WN, winter; SP, spring; SM, summer; AT, autumn) is indicated with an asterisk.

The ratio of oligotroph to copiotroph‐classified ASVs was significantly affected by treatments for both *rrn* copy cut‐off levels, that is, <5 *rrn* copies (*p* = 0.0019), and <2 *rrn* copies (*p* = 0.0044, Table S2). The samples under intermediate and severe water limitations showed a smaller ratio (more oligotroph‐classified ASVs) than the control (Figure 3). However, the difference between treatments was only significant in the second year (2021)—except in summer‐20 at the cut‐off of <5 copies for oligotrophs. The time point significantly affected the copiotroph‐to‐oligotroph ratio (*p* < 0.001, Table S2). Overall, with a cut‐off level of <5, the ratio decreased for all treatments in the first year (2020) as compared to the second (Figure 3A). With a cut‐off level of <2, the ratios remained comparable in the first year (2020), while in the second year, a peak was observed in summer‐21 (Figure 3B).

**FIGURE 4:**
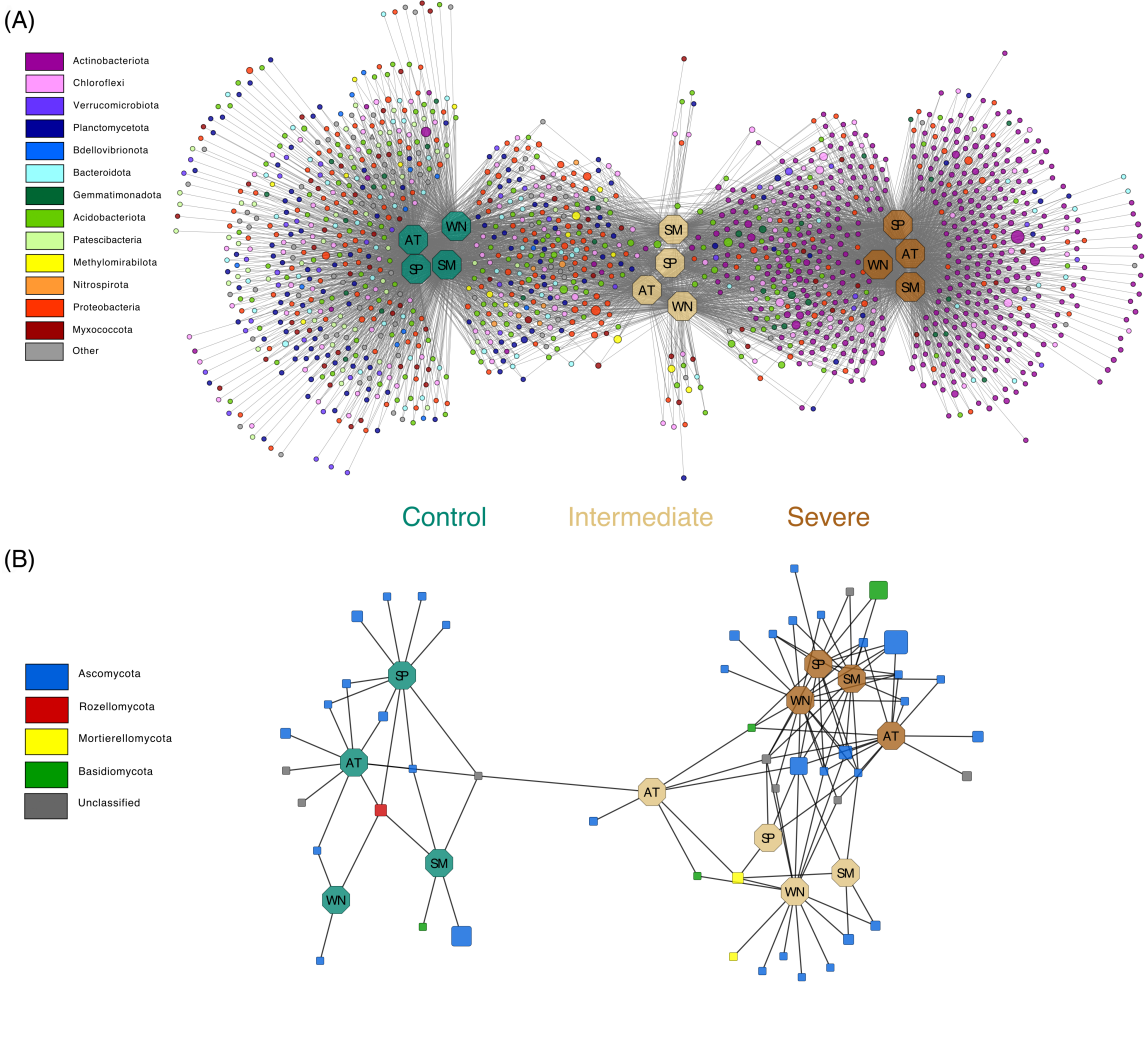
Soil microbial community composition in the second year of the experiment. Prokaryotic (A) and fungal (B) bipartite association networks showing the ASV distribution (circles, prokaryotes; triangles, archaea; squares, fungi) across the different time points (seasons: hexagon; WN, winter; SP, spring; SM, summer; AT, autumn) and treatments. Node sizes are scaled by read counts (square root) and colour‐coded by phylum‐level assignment. Edges correspond to significant associations between ASVs and samples based on indicator species analysis. The edge‐weighted (weighted by ASV association strength) ‘Allegro Fruchterman–Reingold’ algorithm was applied to the network, which clusters samples with higher connectivity (=similar community structure).

As the treatment‐related response was higher in the second year of the experiment (2021), a subset of the dataset was created, and 1453 prokaryotic ASVs with a significant (*q < 0.05*) change in relative abundance were selected for the indicator species analyses and subsequent construction of a bipartite association network. Since only 52 out of 3128 fungal ASVs responded significantly to water deficit (*q* < 0.05) in the whole dataset, a less strict threshold of *q* < 0.1 was applied for the subset, resulting in 50 fungal ASVs used for indicator species analysis and bipartite network construction. The created networks showed a clear separation of the three treatments by clustering but no seasonal time effect (Figure 4). Of the 1453 ASVs in the prokaryotic network, 494 ASVs were unique for the control treatment, 24 for the intermediate, and 338 for the severe treatment. In the fungal network, out of 50 ASVs, 16 were unique for the control treatment, 10 for the intermediate and 15 for the severe treatment. Here, no ASV was shared between the control and severe treatment. After 2 years under severe water limitation, prokaryotic communities were dominated by the phyla Actinobacteriota, Proteobacteria and Chloroflexi, while fungal communities were dominated by Ascomycota and Basidiomycota (Figure 4).

### Soil pH, carbon and nitrogen concentrations

Soil pH was influenced by treatment (*p* = 0.0090, Table S2) and time point (season) (*p* < 0.0001, Table S2). The uppermost pH was measured in Winter‐20, with values ranging between 6.9 and 7.2 (Figure S3A). During the first year (2020), soil pH was lower than in the second year and decreased for all irrigation treatments to 6.8–6.9 (Figure S3A) and remained less acidic in control mesocosms than in mesocosms treated with intermediate and severe water limitations (Figure S3A).

EOC was variable with significant differences across time points (*p* < 0.0001, Table S2). Furthermore, treatments differed within each time point (season) (Figure S3B). EOC was higher under the severe water limitation treatment in summer‐20 and between spring‐21 and autumn‐21, while in the other seasons, higher values were measured for the control treatment (Figure S3B).

C_org_ and TN differed significantly among experimental seasons (*p* < 0.0001, Table S2). C_org_ tended to decrease over the experimental period and significantly differed between the treatments in winter‐21 (Figure S3C). TN concentrations were highest at the beginning of the experiment but then remained lower after spring‐20 (Figure S3D). Soil TN concentrations differed between treatments within each time point (except for spring‐21) and were generally lower in soils treated with water‐limiting conditions compared to the control (Figure S3D).

Soil C:N ratios were significantly affected by time (season) (*p* < 0.0001, Table S2). Overall, soil C:N ratios marginally increased during the experiment (Figure S3E). At the beginning of the irrigation treatments, soil C:N ratios ranged between 16 and 20. The soil C:N ratio increased in the following seasons, with the highest values (between 17 and 29) observed in autumn‐20 (Figure S3E).

Ammonium (NH_4_^+^) and nitrate (NO_3_^−^) concentrations decreased during the experiment and were significantly affected by time (season) (*p* < 0.0001, Table S2). Across all treatments, the highest values were observed in autumn‐20 and winter‐21 (Figure S3F,G).

### Tree development

During both years (2020, 2021), the trees had the greatest radial growth between spring and autumn (*p* < 0.0001, Table S2) and mainly grew in height in spring (*p <* 0.0001, Table S2, Figure 4A,B). The greatest tree height and diameter increments were observed for the control treatment, followed by the intermediate and severe treatments over the whole duration of the study (Figure S4A,B). While tree height and diameter increased in all treatments during the first year (2020), in the second year (2021), no increment was observed for the severe treatment (Figure S4A,B).

The amount of needle fall was highest in autumn, as expected for evergreen pine trees (Figure S4C). However, the difference between treatments in autumn was only significant during the second year (2021), when the control treatment had the highest litter fall compared to water‐limiting conditions.

Living root biomass increased over time for the trees growing under control and intermediate water limitation treatments but not for trees growing under severe water limitation (*p* < 0.0001, Table S2, Figure 4D).

Throughout the experiment, tree photosynthetic assimilation was lowest for trees growing under severe water limitation compared to those growing under the control and intermediate treatments (*p* < 0.0001, Figure S4E, Table S2). In control mesocosms, the highest photosynthetic assimilation was measured in summer‐20, whereas the intermediate and severe mesocosms showed the largest values in spring‐20 (Figure S4E).

### Relationship between microbial communities and tree or soil properties

None of the measured tree parameters directly influenced the composition of the microbial communities. However, changes in soil properties significantly affected the community composition. The composition of prokaryotic and fungal communities was mainly shaped by soil temperature, EOC concentrations, VWC, pH and TN (Tables S4 and S5, Figure S7C,D). Overall, soil temperature was the main driver among the measured properties, followed by EOC concentrations and VWC (Table S5). However, the magnitude of influence was different for both groups (Tables S4 and S5).

## DISCUSSION

### Progressive changes in microbial community structure

Despite the strong influence of the seasonal soil temperature (Tables S4 and S5), the soil water content in our mesocosms and, with that, the water limitation treatments significantly influenced the structure of soil microbial communities (Figure 1, Table S3). This result aligns with our hypothesis that the structure of microbial communities is altered by an extended period of water limitation. The observed changes in community structure appeared progressive rather than circular, with no evident linkage to recurring seasonal trends (Figure 1). For instance, prokaryotic communities showed the highest increase in dissimilarity during winter, spring and summer, which could be related to the increased soil temperatures. Moreover, the strong influence of the seasonal time point could be related to successional changes in tree development. Higher productivity of plants during the main growing season (Figure S4A,B) may have increased the C flux from plants to soils (Bardgett et al., 2005; Wardle et al., 2004). This likely contributed to a higher relative abundance of fungi (Figure S6B) and an increased prokaryotic α‐diversity (Figure S6C), which in turn could explain the increasing dissimilarity of communities. In any case, as communities continued to differentiate independently of seasonal patterns, a more extended period of experimental observations over multiple years would help to identify patterns related to the recurrent seasons and examine if the observed changes are related to successional stages in tree development.

### Increasing dissimilarity of microbial communities under prolonged water limitation

Overall, the prokaryotic soil microbiome was not yet fully adapting to the prolonged water‐limiting conditions, as indicated by the continued further differentiation of the communities between treatments with time (Figure 1). This finding supports our hypothesis that the structure of microbial communities is altered by an extended period of water limitation through continued adaptation to the water‐limiting conditions. This progressive transformation of the microbial community composition, observed in our study, was driven by groups such as Thermoplasmatota and Actinobacteriota, which proliferated early and increased in relative abundance compared to the control over time (Figure 2). At the same time, some tolerant groups, such as Deinococcota and Gemmatimonadota, that began to proliferate only after an extended period of treatments in autumn‐20 contributed to continuous changes in the community structure (Figure 2).

Over 2 years, the degree of dissimilarity between prokaryotic communities increased under prolonged water limitation compared to the control, while fungal communities appeared less affected (Figure 1). This corroborates our initial hypothesis that soil fungi are less sensitive to a prolonged episode of water limitation. Moreover, this finding is in agreement with an earlier shorter‐term study in the same Scots pine mesocosms (Jaeger et al., 2023), demonstrating a contrasting sensitivity of bacterial and fungal communities to water limitation. Moreover, this aligns with other studies, for example, by de Vries et al. (2018) and Ren et al. (2018). The greater tolerance of fungal communities to water limitation (Figure 1) is likely explained by their ability to create and extend exploratory hyphal structures (Barnard et al., 2013; de Vries et al., 2012, 2018; Yuste et al., 2011). The observed higher resilience of fungal communities was potentially driven by the stress tolerance of the phyla Ascomycota and Basidiomycota dominating these communities (Figure 4B). Taxa within these phyla could exhibit a greater tolerance towards osmotic stress and potentially a preference for increased solute concentrations (Araújo et al., 2020). This finding corroborates findings from an irrigation experiment in a Scots pine‐dominated forest published by Hartmann et al. (2017), who observed a stable abundance of Ascomycota and an increase of Basidiomycota under long‐term dry conditions. As the dissimilarity of fungal communities between the control and the severe water limitation treatment increased towards later sampling time points, a longer‐term assessment would be necessary to identify changes within fungal communities and the main factors affecting these changes.

### Benefitting, tolerant and susceptible microbial groups

Since the overall abundance of prokaryotes (and fungi) obtained through copy numbers with qPCR remained stable throughout the experiment (Figure S6A), we conclude that biomass was comparable between treatments. From these patterns, we infer that tolerant and benefitting groups of prokaryotes increasingly outcompeted sensitive groups that performed poorly under water limitation. As a complete discussion of indicator taxa is beyond the scope of this study, only salient examples of indicators are discussed in the following sections.

Based on cluster analysis, three main clusters could be identified (Figure 2). The first cluster consisted of groups that largely benefitted from the water‐limiting conditions and were enriched in relative abundance compared to the control treatment. The phylum Gemmatimonadota increased in abundance compared to the control, especially at later sampling points (Figure 2), indicating a late emergence of this phylum. The distribution of Gemmatimonadota in soil tends to depend on moisture availability as this phylum is commonly found in arid soils, pointing to a potential adaption to low soil moisture (DeBruyn et al., 2011). The phylum Deinococcota responded early to water limitation and could withstand prolonged drying soil conditions (Figure 2). Deinococcota showed a higher abundance under water limitation compared to the control throughout the experiment. This phylum has been described as extremophile, resistant to environmental hazards and abundant in desert soils (Mohammadipanah & Wink, 2016). Their resistance to desiccation through thick and dense carbohydrate coats (Potts, 1994) likely explains the increased abundance of Deinococcota compared to the control in our experiment.

The archaeal phylum Thermoplasmatota also became proportionally more abundant in the water‐limited than in control mesocosms throughout the experiment (Figure 2). Thermoplasmatota are commonly found in extreme environments (Armstrong et al., 2016; Tripathi et al., 2015). Members of these phyla are probably well adapted to survive and possibly thrive in dry soils (Armstrong et al., 2016; Makhalanyane et al., 2013), explaining their significant increase in mesocosms treated with severe water limitation.

After starting the treatments, Actinobacteriota were enriched under water limitation compared to the control (Figure 2). Moreover, Actinobacteriota significantly increased in relative abundance towards later sampling time points (Figures 2 and 4A), which is supported by earlier findings in the same system (Jaeger et al., 2023). We detected many genera belonging to the Actinobacteriota, which increased significantly with prolonged water limitation, such as *Kibella*, *Nakamurella*, *Marmoricola*, *Umezawaea*, *Blastococcus*, *Conexibacter*, *Patulibacter, Solirubrobacter, Gaiella, Actinophytocola*, Lapillicoccus, *Crossiella*, *Rubellimicrobium*, *Parviterribacter*, *Promicromonospora* and *Nocardioides* (Figure S8). Their resistance to dry conditions (Albuquerque et al., 2014; Bastida et al., 2017; Davet, 2004; Mohammadipanah & Wink, 2016; Okoro et al., 2009) could explain the accumulation of these taxa in our study under prolonged water‐limiting conditions (Bachar et al., 2010). An increased proportional abundance of these taxa may be related to some Actinobacteriota (e.g., *Conexibacter*) having rod‐ or coccoid‐shaped cells (Albuquerque & da Costa, 2014; Monciardini et al., 2003). This trait enables them to cope with desiccation, whereas other prokaryotic groups with vegetative hyphae of mycelial are injured rapidly by desiccation (Williams et al., 1972). Therefore, the taxa of Actinobacteriota mentioned above possibly adapt better to severe water limitation and can thus remain sufficiently viable during prolonged episodes of drought and respond quickly to environmental stress (Tóth et al., 2017).

The second cluster consisted of groups partially tolerating water limitation (Figure 2). These groups could tolerate intermediate water limitation (no significant difference from the control) but significantly decreased under severe water limitation compared to the control. For example, the phyla Acidobacteriota, Planctomycetota and Proteobacteria comprising this cluster were highly abundant in our soils. Acidobacteriota are particularly abundant in environments characterised by low resource availability (Fierer et al., 2007; Koyama et al., 2014), indicating a desiccation tolerance and adaption to nutrient‐limited environments (Kielak et al., 2016). This adaptability could explain the observed resistance towards intermediate water‐limiting conditions in our experiment.

Planctomycetota may maintain adaptive traits that increase competitiveness under low soil water contents, as observed during the first year of the experiment (Figure 2). For example, we found a significant increase in the genus *Gemmata* (Figure S8), which was associated with dry soils in a rain‐fall exclusion study in a tropical forest by Bouskill et al. (2013). Moreover, Planctomycetota are known for being slow growers (Kaboré et al., 2020), which might be a strategy to endure nutrient depletion in oligotrophic environments and environmental stresses.

The phylum Proteobacteria was the most abundant in our soils (20.9%) but seemed only to tolerate initial water limitation (Figure 2). Some genera within this phylum increased in abundance under intermediate and severe water limitations. For example, we found a significant increase of the genera *Altererythrobacter*, *Erythrobacter*, *Pseudofulvimonas*, *Sphingomonas, Caenimonas, Rubellimicrobium*, *Roseimicrobium* and *Brevendimonas* (Figure S8). Although individual taxa within the phylum Proteobacteria increased, the overall abundance of this phylum significantly decreased under prolonged severe water limitation (Figure 2). Previous studies have described Proteobacteria as sensitive to drought scenarios (Bouskill et al., 2013; Chodak et al., 2015), supporting our findings. Hartmann et al. (2017) also found an increase of Proteobacteria under irrigation in a forest field site, presumably linked to their copiotrophic lifestyle (Fierer et al., 2007), which could explain their decrease under severe water limitation in our experiment.

The third cluster consisted of groups sensitive to prolonged water limitation, significantly decreasing in both water‐limiting conditions compared to the control (Figure 2). Phyla within this cluster were low in overall relative abundance but taxonomically widespread. For example, the phyla Nitrospirota and Firmicutes and the fungal phylum Roxellomycota were sensitive to prolonged intermediate and severe water limitation (Figure 2).

### Switch from copiotroph‐ to oligotroph‐dominated communities

The availability of EOC provides short‐term C reservoirs for microorganisms because it comprises the most labile and easily degradable fraction of soil organic matter (Haney et al., 2012; Hoffland et al., 2020; Kalbitz et al., 2000). In our experiment, EOC concentrations remained comparable in the first year, even under severe water limitation (Figure S3B). This indicates that the trees may have maintained their root exudation and C allocation belowground—although photosynthetic activity was impaired—as observed by Solly et al. (2023). This might have fueled microbial abundance (Figure S6A,B), specifically the abundance of copiotrophic groups, which preferentially metabolise labile organic C sources. Moreover, higher EOC concentrations could be linked to the release of mucilaginous material by roots to decrease the friction resistance in the drier soils and sustain contact with soil microbes (Fuchslueger et al., 2014; Kakumanu et al., 2013; Walker et al., 2003). Additionally, the higher EOC concentrations observed under severe water limitation during the second year of the experiment (Figure S3B) may have resulted from the cell lysis of dead microbes, as observed by Fuchslueger et al. (2014).

Nevertheless, because the overall abundance of prokaryotes (and fungi) remained stable throughout the experiment (Figure S6A), it is more likely that higher EOC concentrations resulted from impaired C uptake by microbes through a lack of water than from cell lysis. Thus, the observed pattern might relate to a shift towards oligotrophic communities—as we had hypothesised—which have less affinity to consume EOC. Dry conditions can rapidly alter microbial communities towards oligotrophic groups, which can survive with alternative types and availabilities of C resources (Aldén et al., 2001; Drenovsky et al., 2004; Soong et al., 2020).

We observed a lower copiotroph‐to‐oligotroph ratio under decreased soil moisture and reduced photosynthetic activity of the young trees (Figure 3). A lower ratio under intermediate and severe water limitation indicates a preferential proliferation of oligotrophic prokaryotes, which supports our hypothesis of an induced shift in microbial communities towards oligotrophy. Using the *rrn* operon database to classify oligotrophic groups, Acidobacteriota had a mean copy number of 1.1, Chloroflexi of 1.6, Crenarchaeota of 1.7 and Verrucomicrobiota of 2.5. With this, all of these were classified as oligotrophs in our study, which matches the existing literature (Fierer et al., 2007; Martens‐Habbena et al., 2009; Trivedi et al., 2018). Moreover, we found quantitatively more ASVs associated with the mentioned phyla in the second year under severe water limitation (Figure 4).

Our observation aligns with findings from Pascault et al. (2013) and Hartmann et al. (2017), who observed a reduction in oligotrophic groups and a related increase of more generalist copiotrophs under nutrient‐rich conditions with greater soil moisture levels. Since the difference in the ratio between treatments was only significant during the second year of the experiment (Figure 3), the switch from copiotroph‐ to oligotroph lifestyle within communities appears to be an effect of prolonged water deficit and might largely be attributed to the general slow proliferation times of oligotrophs (Fierer et al., 2007; Naylor & Coleman‐Derr, 2018; Trivedi et al., 2013).

Overall, the shift from copiotroph‐ to oligotroph‐dominated communities might also point to a slowdown of the C turnover rate under prolonged water limitation.

### Increase of saprotrophic and ligninolytic taxa

We hypothesised that an alteration in substrate quality with more recalcitrant C resources would increase the abundance of saprotrophic and ligninolytic taxa. During the second year of the study, we observed a reduced amount of living root biomass under severe water limitation (Figure S4D), leading most likely to an increased input of dead plant material (i.e., dead roots) to the soil. In parallel, we observed an increase in the soil C:N ratio (Figure S3E), which is in line with the C:N ratio reported for the top‐soil layer in studies from the same field site (e.g., Hartmann et al., 2017; Herzog et al., 2014; Solly et al., 2018). The increasing C:N ratio generally indicates an accumulation of complex organic compounds—such as lignin and suberin—which favours microbes with the ability to decompose recalcitrant structures (Baldrian et al., 2012; Lindahl & Tunlid, 2015; Van der Wal et al., 2013; Žifčáková et al., 2017). Fine roots are known as the dominant source of recalcitrant plant litter in hardwood forests (Xia et al., 2015); also, the input of needle litter—which was sustained in autumn under all treatments (Figure S4C)—might have contributed to an accumulation of complex plant‐derived organic compounds in the mesocosms.

This accumulation has likely favoured the abundance of saprotrophic fungal groups and ligninolytic bacterial taxa, supporting our hypotheses and corroborating earlier findings in the same system (Jaeger et al., 2023). For example, we found that the abundance of *Ilyonectria* increased under prolonged water deficit (Figure S8), of which species have been associated with the decay of plant roots and wood (Cabral et al., 2012; Siles et al., 2018). Furthermore, *Cladosporium* significantly increased under intermediate and severe water limitations (Figure S8). Although described as predominantly epiphytic with preferences for the leaf interior and exterior (Flessa et al., 2012; Khan et al., 2016), *Cladosporium* is commonly found on dead plant material and considered pathogenic (Klironomos et al., 1997).

For bacteria, ligninolytic capabilities have mainly been reported for Actinobacteriota (Abdel‐Hamid et al., 2013), known as degraders of plant material in soils (Sanaullah et al., 2016). For example, members of the genera *Promicromonospora*, *Conexibacter* and *Solirubrobacter* are involved in the degradation of lignin and lignocellulose (López‐Mondéjar et al., 2019; Wilhelm et al., 2019) of which we observed an increase in relative abundance under prolonged water limitation (Figure S8). Actinobacteriota were highly abundant in a study about root litter decomposition in a drought‐prone Scots pine forest stand by Herzog et al. (2019). Therefore, the observed increase of Actinobacteriota under severe water limitation in our study (Figures 2 and 4A) might also point to increased amounts of dead root material.

Additionally, some groups of Proteobacteria might be capable of degrading cellulose and hemicellulose from plant biomass (López‐Mondéjar et al., 2016). For example, the genera *Luteimonas* and *Sphingomonas* increased under prolonged water limitation (Figure S8), of which some species are ligninolytic, capable of degrading aromatic structures (Masai et al., 1999).

### Potential consequences for tree development

#### Decrease of symbiotic and N‐cycling taxa

We observed lower aboveground productivity (height and stem increment) under severe water limitation, especially during the second year of the experiment (Figure S4A,B). The decreased abundance of symbiotic and N‐cycling taxa from various phyla with lower soil moisture might have impaired the water and nutrient availability for young trees, which, in addition to the direct effects of water limitation on trees, can partially explain the reduction of development and growth under extended water‐limiting conditions.

Although fungi appeared less affected by prolonged water limitation, our findings reveal significant changes in the abundance of symbiotic taxa associated with changing physicochemical soil properties under drier conditions. For example, the genera *Umbelopsis* (Mucoromycota*)* forming mutualistic associations with plants (van der Heijden et al., 2015) significantly decreased under prolonged intermediate and severe water deficits (Figure S8). We also observed a decrease of symbiotic taxa belonging to the Ascomycota, although the phylum appeared tolerant towards prolonged water deficit. For example, the family Tuberaceae, known as truffles, forms symbiotic associations with plants (Trappe et al., 2009), and decreased in mesocosm with water limitation, supported by previous findings (Jaeger et al., 2023). Furthermore, the family Tulasnella, which forms mycorrhizae with worldwide distribution in various ecosystems (Freitas et al., 2020), decreased with a prolonged water deficit.

In soils, ammonium is quickly transformed into plant‐available nitrate by *Nitrospira* (Nitrospirota) (Clark et al., 2021). Thus, a decrease in the genus *Nitrospira*, as observed in this study (Figure S8), might have impaired the nitrogen availability for the young trees. Furthermore, *Hyphomicrobium* (Proteobacteria) was negatively affected by water limitation (Figure S8), thus potentially limiting nutrient availability through a reduction in N‐fixation (Lladó et al., 2017; Oren & Xu, 2014). We also observed a decrease in the endophytic genus *Paenibacillus* (Firmicutes) under extended water limitation (Figure S8). *Paenibacillus* is known to improve the nutrient status of plants through phosphate solubilisation and N‐fixation under dry and nutrient‐poor conditions (Bal et al., 2012; Puri et al., 2018). Species of the genus *Flavobacterium* (Bacteroidetes) are also known to fix N in the phyllosphere of plants (Giri & Pati, 2004; Kämpfer et al., 2015); because this genus decreased (Figure S8) this might as well point to a potential decrease of N‐fixing activities under prolonged water limitation. Some strains of the decreasing genus *Erythrobacter* (Proteobacteria) exhibit a denitrifying activity (Shioi et al., 1988; Zheng et al., 2016) and thus might also have impaired N‐cycling under prolonged water limitation.

The recognised decrease of potential N‐fixing and N‐cycling bacteria likely supports our finding of reduced plant fitness (growth and photosynthesis) due to reduced N‐cycling under prolonged water limitation.

## CONCLUSION

Our study reveals that prolonged water limitation progressively increases the distinction of prokaryotic microbial communities compared to the well‐watered control. Our findings further suggest that the structure of prokaryotic communities progressively adapts to low levels of soil moisture during long‐lasting episodes of water limitation. Moreover, our results show that fungal communities are less affected by prolonged water limitation than prokaryotic communities. Despite a strong influence of the time point and associated changes in soil temperatures, fungal and prokaryotic communities continued to differentiate independently of the recurrent seasons. The presented results further indicate that although water stress does not alter the total abundance and biomass of soil microorganisms, it shifts the composition of microbial communities towards desiccation‐tolerant groups that outcompete less adapted groups. Specifically, the abundance of saprotrophic groups progressively increased alongside an accumulation of dead plant material, and the abundance of known symbiotic and N‐cycling taxa decreased under experimental drought, likely affecting the development of the young trees. Additionally, we could show that the lifestyle of prokaryotic taxa shifts from copiotrophic to oligotrophic under prolonged water limitation, which suggests a slowdown in the C turnover rate. Overall, these findings contribute to our understanding of how soil microbial communities adapt to different thresholds of water limitation and demonstrate that prolonged episodes of drought will progressively alter the structure of microbial communities with potential losses of critical functions provided by the soil microbiome.

## AUTHOR CONTRIBUTIONS

**Astrid C. H. Jaeger:** Conceptualization (equal); data curation (lead); formal analysis (lead); investigation (lead); methodology (equal); software (equal); visualization (lead); writing – original draft (lead); writing – review and editing (equal). **Martin Hartmann:** Conceptualization (equal); data curation (equal); formal analysis (equal); investigation (equal); methodology (equal); project administration (supporting); software (lead); supervision (equal); visualization (equal); writing – original draft (supporting); writing – review and editing (equal). **Rafaela Feola Conz:** Investigation (supporting); methodology (equal); writing – review and editing (equal). **Johan Six:** Conceptualization (equal); resources (lead); supervision (supporting); writing – review and editing (equal). **Emily F. Solly:** Conceptualization (equal); data curation (equal); funding acquisition (lead); investigation (equal); methodology (equal); project administration (lead); supervision (lead); writing – original draft (supporting); writing – review and editing (equal).

## CONFLICT OF INTEREST STATEMENT

The authors declare no conflicts of interest.

## Supporting information


**DATA S1:** Supporting Information.

## Data Availability

The sequence data of this study have been submitted to the European Nucleotide Archive (ENA) at EMBL‐EBI (http://www.ebi.ac.uk/) under accession numbers PRJEB53192 and PRJEB61158. Meta data that support the findings of this study are available from the corresponding author upon reasonable request.
