# Supplementary material for: Prolonged water limitation shifts the soil microbiome from copiotrophic to oligotrophic lifestyles in Scots pine mesocosms
Source: Environ Microbiol Rep. 2023 Nov 22;16(1):e13211. doi: 10.1111/1758-2229.13211 (PMC10866073; doi:10.1111/1758-2229.13211)
Supplement: Supplementary file 1 — DATA S1: Supporting Information. [file EMI4-16-e13211-s001.docx]

Supplementary Material


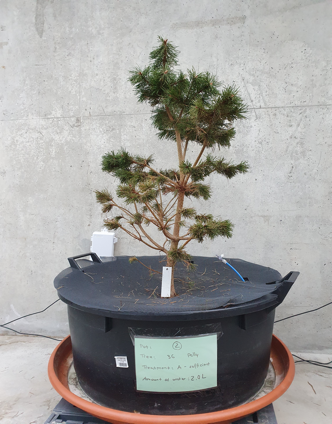

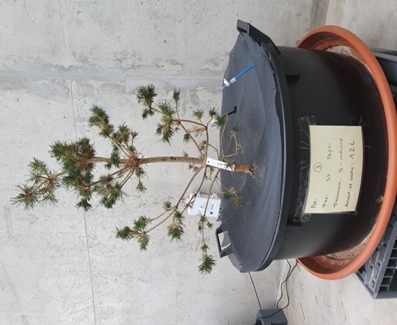

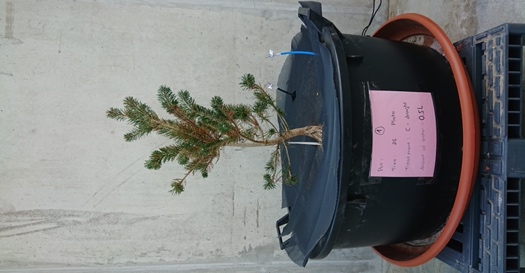

**Supplementary Figure 2** **Volumetric water content across treatments in each season.** Mean volumetric water content (VWC) in % for each treatment (n=6) in all sampled seasons (n=8), color scheme as indicated in previous figures. Error bars indicate the standard error of the mean. Small letters indicate significant differences between groups based on estimated marginal means of linear mixed effect models.

**Supplementary Figure 1 Experimental set-up of the Scots pine soil mesocosms in the greenhouse.** Set-up of the experiment with a total of 18 mesocosms treated with three different levels of irrigation (control, intermediate, severe).

**Supplementary Figure 3** **Overview of** **seasonal changes in soil properties.** Mean soil pH (A), mean extractable organic carbon per gram dry weight soil (B), mean organic carbon concentrations in % (C), mean total nitrogen concentrations in % (D), mean C:N ratio (E), mean ammonium concentrations per gram dry weight soil (F), mean soil nitrate concentrations per gram dry weight soil (G) for each treatment group across time points (season), displayed with standard error. Letters indicate differences between time points (season) obtained by estimated marginal means of linear mixed effect models. Statistical significance (p < 0.05) of planned contrasts between treatments within each time point (season) is indicated with an asterisk.

**Supplementary Figure 4** **Measured plant parameters for each treatment across seasons.** Mean of seasonal increment in tree height since the set-up (A), increment in tree diameter since the set-up (B), amount of collected needle litter as dry weight (C), amount of dry weight living root biomass (D), measured photosynthesis (E), displayed with standard error of the mean. Letters indicate differences between time points (seasons) obtained by estimated marginal means of linear mixed effect models. Statistical significance (p < 0.05) of planned contrasts between treatments within each time point (season) is indicated with an asterisk.

**Supplementary Figure 5** **Rarefaction curves across all samples for prokaryotes (A) and fungi (B)**.

**Supplementary Figure 6** **Abundance and α-diversity of prokaryotic and fungal communities.** Estimated copies of the prokaryotic 16S rRNA gene (A) and the fungal 18S rRNA gene (B) assessed for each treatment across time with qPCR assays. The α-diversity examined by observed richness, Pielou's evenness, and Shannon diversity for each treatment group across time (seasons) for prokaryotes (C-E) and fungi (F-H). Means are displayed with error bars indicating the standard error of the mean. Capital letters indicate significant differences between time points (season) obtained by estimated marginal means of linear mixed effect models. An asterisk indicates the statistical significance (p < 0.05) of planned contrasts between treatments within a time point (season).

**Supplementary Figure 7** **Soil microbial community structure and influence of soil properties.** Unconstrained principal coordinate analysis (PCO) ordinations of prokaryotic (A) and fungal (B) communities based on Bray-Curtis dissimilarities calculated from ASV abundances. Distance-based redundancy analysis (dbRDA) showing the significant (p < 0.05) relationship between prokaryotic (C) and fungal (D) community structure with the measured physicochemical soil properties.

**Supplementary Figure 8 Change in the relative abundance of indicator taxa**. Relative change (z-transformed) in abundance of bacterial and fungal genera under intermediate and severe water deficit compared to the control treatment. The first vertical panel represents the relative change in abundance from the overall mean, including the average change (circles) and the corresponding standard error (horizontal lines)—only genera with significant (q < 0.05) change in abundance are displayed. The second vertical panel represents the relative abundance of the genera (horizontal grey bars) as obtained by read counts.

| **Season** | **Winter-20** | **Spring-20** | **Summer-20** | **Autumn-20** | **Winter-21** | **Spring-21** | **Summer-21** | **Autumn-21** |
| --- | --- | --- | --- | --- | --- | --- | --- | --- |
| **Sampling Date** | 13.01.2020 | 11.05.2020 | 27.07.2020 | 19.10.2020 | 25.01.2021 | 03.05.2021 | 12.07.2021 | 06.09.2021 |
| **Temp. GH [°C]** | 10.3 ± 0.2 | 16.5 ± 0.1 | 21.5 ± 0.1 | 15.9 ± 0.1 | 10.3 ± 0.1 | 16.1 ± 0.2 | 22.6 ± 0.4 | 21.1 ± 0.3 |
| **Temp. Soil [°C]** | 9.1 ± 0.1 | 17.5 ± 0.1 | 21.9 ± 0.1 | 14.5 ± 0.1 | 9.4 ± 0.1 | 15.9 ± 0.6 | 23.8 ± 0.8 | 21.9 ± 0.9 |
| **Humidity GH [%]** | 52.9 ± 1.7 | 46.9 ± 0.3 | 64.3 ± 0.8 | 65.9 ± 0.5 | 51.0 ± 0.7 | 41.2 ± 0.1 | 61.8 ± 0.8 | 56.1 ± 3.1 |

**Supplementary Table 1 Overview of the greenhouse conditions.** Eight sampling dates across the seasons Winter-20, Spring-20, Summer-20, Autumn-20, Winter-21, Spring-21, Summer-21, and Autumn-21. Mean and standard deviation of the greenhouse temperature (Temp. GH), the greenhouse humidity (Humidity GH), and the soil temperature (Temp. Soil) for every season.

|  | **Treatment (T)** | | **Season (S)** | | **T x S** | |
| --- | --- | --- | --- | --- | --- | --- |
| **Parameter** | **F** | **P** | **F** | **P** | **F** | **P** |
| VWC | 103.00 | **<.0001** | 92.00 | **<.0001** | 14.00 | **<.0001** |
| pH | 6.00 | **0.0090** | 27.00 | **<.0001** | 1.00 | 0.2314 |
| C:N | 0.00 | 0.6565 | 8.00 | **<.0001** | 1.00 | 0.5329 |
| TN | 1.00 | 0.2632 | 18.00 | **<.0001** | 1.00 | 0.6855 |
| NH_4_^+^ | 6.60 | **0.0074** | 18.50 | **<.0001** | 0.80 | 0.7138 |
| NO_3_^-^ | 0.72 | 0.5031 | 40.33 | **<.0001** | 2.46 | **0.0050** |
| EOC | 3.00 | 0.0782 | 26.00 | **<.0001** | 6.00 | **<.0001** |
| C_org_ | 0.00 | 0.6745 | 7.00 | **<.0001** | 1.00 | 0.1987 |
| 16S gene copies | 0.00 | 0.8700 | 7.00 | **<.0001** | 1.00 | 0.8600 |
| 18S gene copies | 1.00 | 0.2900 | 6.00 | **<.0001** | 0.00 | 0.9600 |
| Copiotroph:Oligotroph <5 | 9.25 | **0.0019** | 6.58 | **<.0001** | 3.50 | **0.0001** |
| Copiotroph:Oligotroph <2 | 7.59 | **0.0044** | 5.65 | **<.0001** | 4.34 | **<.0001** |
| Height growth | 2.16 | 0.1459 | 58.16 | **<.0001** | 6.88 | **<.0001** |
| Radial growth | 9.22 | **0.0019** | 18.87 | **<.0001** | 4.85 | **<.0001** |
| Root biomass | 0.80 | 0.4594 | 8.00 | **<.0001** | 2.20 | **0.0383** |
| Photosynthesis | 93.94 | **<.0001** | 13.58 | **<.0001** | 4.07 | **0.0007** |
| Needle litter | 1.20 | 0.3246 | 60.80 | **<.0001** | 2.50 | **0.0045** |

**Supplementary Table 2** **Effects of irrigation treatment and seasonal time points on different soil properties and tree parameters.** Effects of treatment (T, n=3), season (S, n=8) their interaction (TxS, n=24) on soil physicochemical parameters, abundance of taxonomic markers (16S rRNA gene, 18S rRNA gene), copiotroph to oligotroph ratios, and tree parameters were tested with linear mixed effect models (lme) and displayed with the F-ratio (F) and level of significance (p-value). Significant results are indicated with bold numbers.

**Supplementary Table 3** **Effects of irrigation treatment and seasonal time point on soil microbial α-diversity and β-diversity**. Effects of treatment (T, n=3), season (S, n=8), and their interaction (TxS, n=24) on prokaryotic and fungal α-diversity and β-diversity assessed by univariate (α-diversity) and multivariate (β-diversity) permutational analysis of variance (PERMANOVA). Values indicate the F-ratio (F), the level of significance (P), and the explained variance (R^2^). Significant heterogeneities of variance assessed by permutational analysis of univariate dispersion (PERMDISP) are indicated with an asterisk.

| **Prokaryotes** | **⍺-Diversity** | | | | | | | | |  | **β-Diversity** | | |
| --- | --- | --- | --- | --- | --- | --- | --- | --- | --- | --- | --- | --- | --- |
|  | **Observed Richness (S)** | | | **Pielou's Evenness (J)** | | | **Shannon Diversity (H)** | | |  | **Bray-Curtis dissimilarity** | | |
|  | R^2^ | F | P | R^2^ | F | P | R^2^ | F | P |  | R^2^ | F | P |
| Treatment (T) | 0.032 | 5.220 | **0.002*** | 0.063 | 21.212 | **0.001*** | 0.055 | 14.202 | **0.001*** |  | 0.061 | 5.141 | **0.001*** |
| Season (S) | 0.547 | 25.357 | **0.001*** | 0.682 | 65.907 | **0.001*** | 0.645 | 47.620 | **0.001*** |  | 0.132 | 3.190 | **0.001*** |
| T x S | 0.053 | 1.236 | 0.237 | 0.080 | 3.859 | **0.001** | 0.070 | 2.584 | **0.003*** |  | 0.105 | 1.273 | **0.001*** |

| **Fungi** | **⍺-Diversity** | | | | | | | | |  | **β-Diversity** | | |
| --- | --- | --- | --- | --- | --- | --- | --- | --- | --- | --- | --- | --- | --- |
|  | **Observed Richness (S)** | | | **Pielou's Evenness (J)** | | | **Shannon Diversity (H)** | | |  | **Bray-Curtis dissimilarity** | | |
|  | R^2^ | F | P | R^2^ | F | P | R^2^ | F | P |  | R^2^ | F | P |
| Treatment (T) | 0.060 | 9.685 | **0.001** | 0.016 | 1.185 | 0.336 | 0.032 | 2.795 | **0.048** |  | 0.031 | 2.527 | **0.001** |
| Season (S) | 0.495 | 22.881 | **0.001*** | 0.129 | 2.803 | **0.006** | 0.191 | 4.772 | **0.001** |  | 0.147 | 3.420 | **0.001** |
| T x S | 0.077 | 1.789 | **0.048*** | 0.075 | 0.818 | 0.688 | 0.099 | 1.236 | 0.229 |  | 0.091 | 1.054 | 0.153 |

|  | **β-Diversity (Bray-Curtis Dissimilarity)** | | | | | | |
| --- | --- | --- | --- | --- | --- | --- | --- |
|  | **Prokaryotes** | | |  | **Fungi** | | |
|  | **R^2^** | **F** | **P** |  | **R^2^** | **F** | **P** |
| VWC | 0.007 | 1.081 | 0.209 |  | 0.011 | 1.682 | **0.009** |
| pH | 0.014 | 2.063 | **0.001** |  | 0.012 | 1.729 | **0.013** |
| C:N | 0.008 | 1.166 | 0.140 |  | 0.008 | 1.225 | 0.152 |
| C_org_ | 0.008 | 1.124 | 0.177 |  | 0.009 | 1.290 | 0.098 |
| TN | 0.012 | 1.758 | **0.010** |  | 0.007 | 1.072 | 0.278 |
| EOC | 0.012 | 1.819 | **0.002** |  | 0.015 | 2.235 | **0.007** |
| NH_4_^+^ | 0.009 | 1.329 | **0.045** |  | 0.007 | 0.999 | 0.443 |
| NO_3_^-^ | 0.007 | 1.050 | 0.271 |  | 0.010 | 1.416 | 0.051 |
| Height growth | 0.008 | 1.146 | 0.136 |  | 0.019 | 2.745 | 0.076 |
| Radial growth | 0.007 | 1.005 | 0.397 |  | 0.009 | 1.327 | 0.822 |
| Needle litter | 0.007 | 1.067 | 0.269 |  | 0.006 | 0.822 | 0.193 |
| Soil Temp. | 0.020 | 3.017 | **0.001** |  | 0.008 | 1.178 | **0.001** |

**Supplementary Table 4** **Effects of measured soil and tree parameters on the prokaryotic and fungal β-Diversity**. Effects assessed by univariate permutational analysis of variance (PERMANOVA). Values indicate the F-ratio (F), the level of significance (P), and the explained variance (R^2^). Significant results are indicated with bold numbers.

**Supplementary Table 5** **Effects of physicochemical soil properties on soil microbial community structure** based on building a parsimony model for variable selection with the function ordistep followed by PERMANOVA, displayed with F-ratio (F) and level of significance (P).

| **Prokaryotes** | | | |  | **Fungi** | | | |
| --- | --- | --- | --- | --- | --- | --- | --- | --- |
|  | R^2^ | **F** | **P** |  |  | R^2^ | **F** | **P** |
| Soil Temp. | 0.019 | 2.757 | 0.001 |  | Soil Temp. | 0.017 | 2.536 | 0.001 |
| EOC | 0.015 | 2.219 | 0.001 |  | EOC | 0.015 | 2.224 | 0.009 |
| VWC | 0.012 | 1.750 | 0.005 |  | VWC | 0.015 | 2.145 | 0.002 |
| pH | 0.012 | 1.747 | 0.003 |  | pH | 0.013 | 1.860 | 0.006 |
| TN | 0.009 | 1.262 | 0.066 |  | TN | 0.010 | 1.485 | 0.032 |
